# *Ziziphus nummularia* Attenuates the Malignant Phenotype of Human Pancreatic Cancer Cells: Role of ROS

**DOI:** 10.3390/molecules26144295

**Published:** 2021-07-15

**Authors:** Joelle Mesmar, Manal M. Fardoun, Rola Abdallah, Yusra Al Dhaheri, Hadi M. Yassine, Rabah Iratni, Adnan Badran, Ali H. Eid, Elias Baydoun

**Affiliations:** 1 Department of Biology, Faculty of Arts and Sciences, American University of Beirut, Beirut P.O. Box 11-0236, Lebanon; jm104@aub.edu.lb (J.M.); mmf27@mail.aub.edu (M.M.F.); rha62@mail.aub.edu (R.A.); 2 Department of Biology, College of Science, United Arab Emirates University, Al-Ain P.O. Box 15551, United Arab Emirates; yusra.aldhaheri@uaeu.ac.ae (Y.A.D.); r_iratni@uaeu.ac.ae (R.I.); 3 Biomedical Research Center, Qatar University, Doha P.O. Box 2713, Qatar; hyassine@qu.edu.qa; 4 Department of Basic Sciences, University of Petra, Amman P.O. Box 961343, Jordan; abadran@uop.edu.jo; 5 Department of Basic Medical Sciences, College of Medicine, QU Health, Qatar University, Doha P.O. Box 2713, Qatar; 6 Biomedical and Pharmaceutical Research Unit, QU Health, Qatar University, Doha P.O. Box 2713, Qatar

**Keywords:** herbal medicine, pancreatic cancer, malignant phenotype, ROS, *Ziziphus nummularia*

## Abstract

Pancreatic cancer (PC) is the fourth leading cause of all cancer-related deaths. Despite major improvements in treating PC, low survival rate remains a major challenge, indicating the need for alternative approaches, including herbal medicine. Among medicinal plants is *Ziziphus nummularia* (family *Rhamnaceae*), which is a thorny shrub rich in bioactive molecules. Leaves of *Ziziphus nummularia* have been used to treat many pathological conditions, including cancer. However, their effects on human PC are still unknown. Here, we show that the treatment of human pancreatic ductal adenocarcinoma cells (Capan-2) with *Ziziphus nummularia* ethanolic extract (ZNE) (100–300 μg/mL) attenuated cell proliferation in a time- and concentration-dependent manner. Pretreatment with N-acetylcysteine, an ROS scavenger, attenuated the anti-proliferative effect of ZNE. In addition, ZNE significantly decreased the migratory and invasive capacity of Capan-2 with a concomitant downregulation of integrin α_2_ and increased cell–cell aggregation. In addition, ZNE inhibited in ovo angiogenesis as well as reduced VEGF and nitric oxide levels. Furthermore, ZNE downregulated the ERK1/2 and NF-κB signaling pathways, which are known to drive tumorigenic and metastatic events. Taken together, our results suggest that ZNE can attenuate the malignant phenotype of Capan-2 by inhibiting hallmarks of PC. Our data also provide evidence for the potential anticancer effect of *Ziziphus nummularia*, which may represent a new resource of novel anticancer compounds, especially ones that can be utilized for the management of PC.

## 1. Introduction

Cancer is the second leading cause of death worldwide, accounting for 13% of global mortalities [1]. In 2018, 9.6 million people died from cancer [1], with pancreatic cancer (PC) being the fourth leading cause of cancer-related deaths [2]. Despite great advancements in its management and treatment, PC continues to exhibit a low survival rate with 95% of cases being incurable [3,4]. This low rate is largely due to delayed diagnosis as well as resistance to therapies [5,6]. This prompts the need for alternative approaches such as herbal medicine. Relevantly, the use of herbs and plants for the treatment of several diseases including cancer is receiving increasing interest due to its low cost, somewhat more tolerable side effects, and more acceptance among patients, in addition to evidence from folk medicine and accumulated wisdom of previous generations [7].

*Ziziphus nummularia*, commonly known as Sidr, is a branched thorny shrub belonging to the family Rhamnaceae [8]. It grows largely in arid and dry areas [9], and it has been used in folk medicine [10,11]. Since it is bountiful in bioactive molecules such as tannins, flavonoids, steroids, glycosides, and alkaloids, *Ziziphus nummularia* has been used to treat a wide spectrum of pathological conditions including cold, diarrhea, anemia, bronchitis, indigestion, and inflammation [12]. In addition, its extracts are well known to inhibit the malignant phenotype of several cancer types, such as human breast cancer (MCF-7), leukemia (K-562), ovarian cancer (OVCAR-3), human colon adenocarcinoma (HT-29), and human kidney carcinoma (A-498) [13,14]. In vivo, a chemical extracted from *Z. nummularia* decreased the tumor volume of Ehrlich ascites carcinoma-bearing mice, and more importantly, it increased their survival [13]. These studies suggest that *Z. nummularia* may have a beneficial effect in the fight against cancer, which prompted us to test the effect of this shrub on PC, which is a rather formidable and unpreventable type of cancer [15].

In the present study, we examined the effect of the *Z. nummularia* ethanolic extract (ZNE) on the malignant phenotype of human pancreatic ductal adenocarcinoma cells (Capan-2). We tested the effects of ZNE on the proliferation, migration, adhesion, aggregation, and invasion of Capan-2. In addition, we assessed whether ZNE modulates in ovo angiogenesis using a chorioallantoic membrane assay. To mechanistically determine the basis of ZNE effects, we also assessed the phosphorylation of ERK1/2 activity, α_2_ integrin expression, and VEGF levels. Moreover, the roles of ROS and NF-κB in the ZNE-modulated proliferation of Capan-2 cells were tested.

## 2. Results

### 2.1. ZNE Inhibits the Proliferation of Capan-2 Pancreatic Cancer Cells

**Figure 1:**
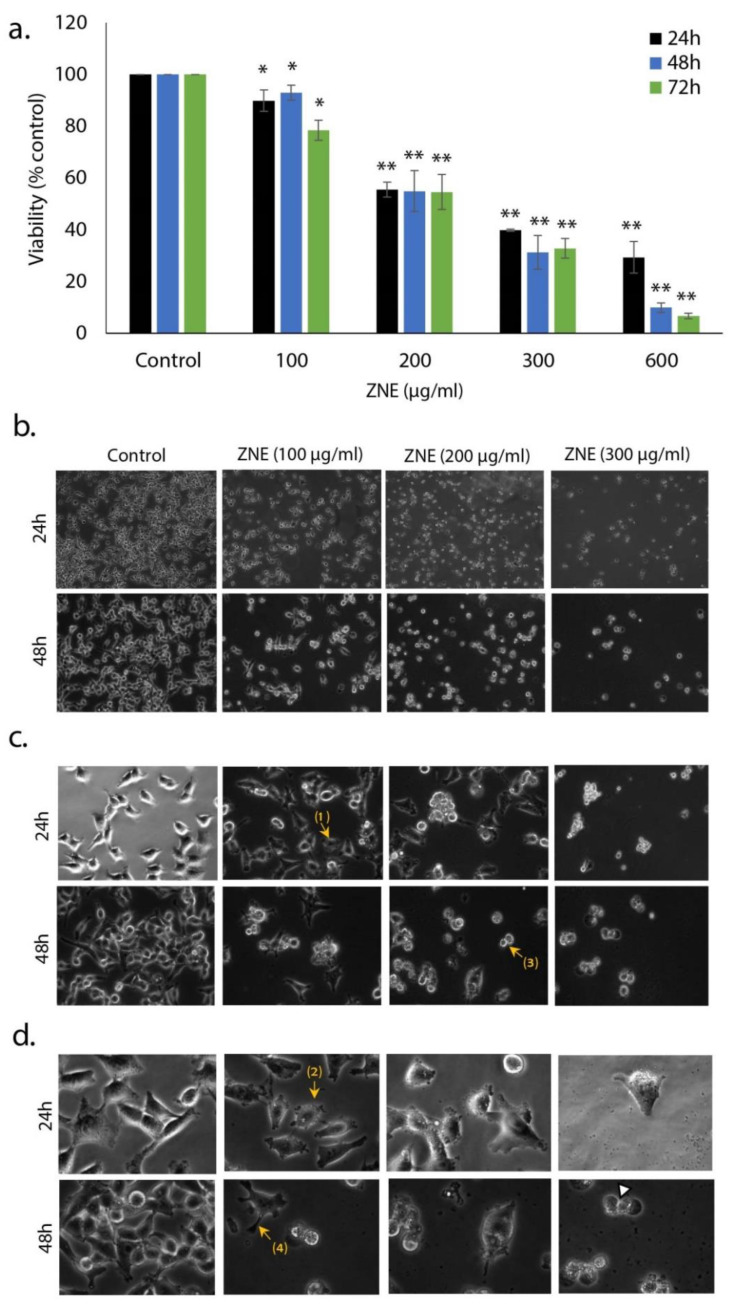
**Inhibition of cellular viability by *Ziziphus nummularia* ethanolic extract.** (**a**) Capan-2 cancer cells were treated with and without the indicated concentrations of *Ziziphus nummularia* ethanolic extract (ZNE) for 24, 48, and 72 h. Cell viability was carried using the metabolic-dye-based MTT assay, as described in Materials and Methods. Data represent the mean of three independent experiments performed in triplicate. Morphological changes were observed at magnifications of 4× (**b**), 10× (**c**), or 40× (**d**). Arrows show (1) cell shrinkage, (2) membrane blebbing, (3) apoptotic bodies, and (4) echinoid spikes. * denotes *p* < 0.05, and ** *p* < 0.01.

To examine the anti-proliferative activity of *Ziziphus nummularia* ethanolic extract (ZNE) on PC cells, the effect of various concentrations (0, 100, 200, 300, and 600 μg/mL) on the viability of the Capan-2 cell line was assessed (Figure 1a). The results show that ZNE treatment decreased cell viability in a concentration-dependent manner. For instance, cell viability was 92.9 ± 2.8, 54.9 ± 7.8, 31.3 ± 6.5, or 1.8 ± 2.8% that of control after treatment with 100, 200, 300, or 600 μg/mL of ZNE, respectively. The IC_50_ (half maximal inhibitory concentration) was 251, 217, and 209 μg/mL at 24, 48, and 72 h, respectively.

Morphological observation of ZNE-treated cells not only showed a concentration-dependent decrease in the number of cells per microscopic field but also that ZNE-treated cells exhibited features characteristic of apoptosis (Figure 1b). Indeed, at higher magnifications, cells showed cytoplasmic shrinkage, rounded shape, loss of epithelial morphology, and appearance of membrane blebbing and apoptotic bodies (Figure 1c,d).

### 2.2. ZNE Induced Caspase-3-Dependent Apoptosis in Capan-2 Cells

**Figure 2:**
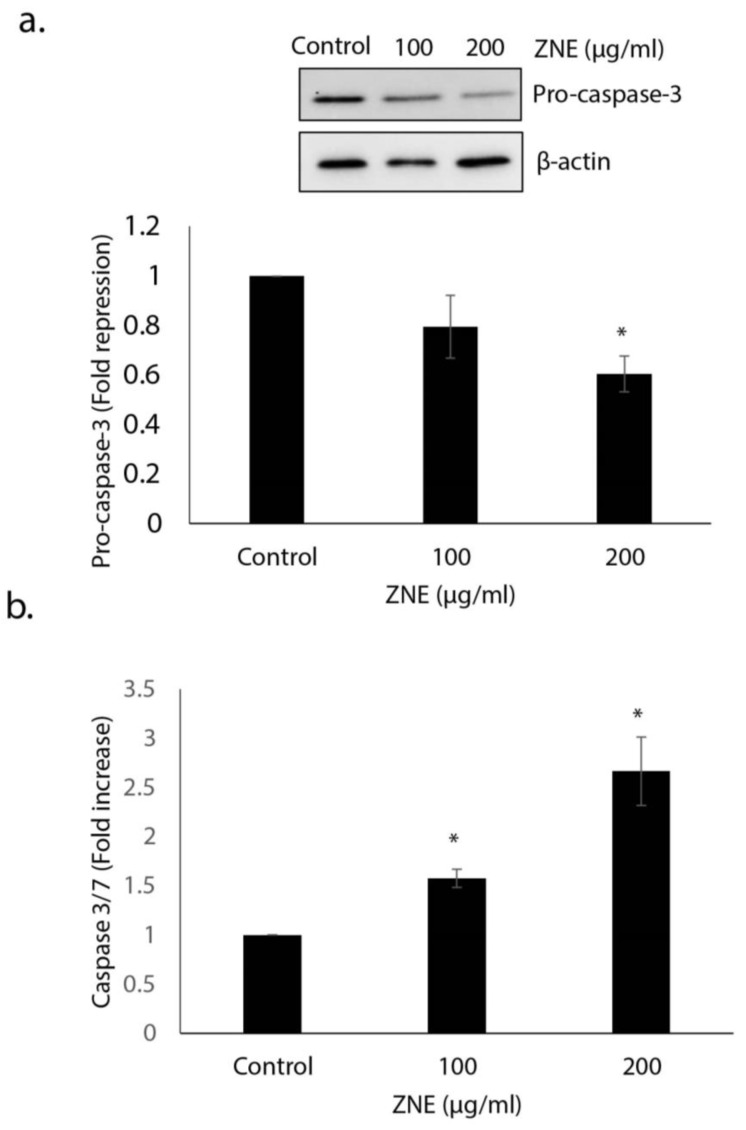
***Ziziphus nummularia* induces apoptosis in Capan-2 cancer cells**. (**a**) Cells were treated for 48 h with or without the indicated concentrations of ZNE. The expression levels of procaspase-3 were determined by Western blotting and β-actin used as loading control. The blot shown is representative of three independent experiments. (**b**) Caspase 3/7 activity was assessed 48 h after treatment of Capan-2 cells with ZNE (100 and 200 μg/mL) or ethanol (control) cells. The relative caspase 3/7 activity was normalized to the number of viable cells per well and is expressed as fold of induction compared to the untreated control cells. * denotes *p* < 0.05.

Having shown that ZNE induces morphological changes characteristic of apoptosis, we next sought to confirm that apoptosis is indeed activated in Capan-2 cells. Toward this, we first examined the protein level of procaspase-3, which upon activation of apoptosis becomes cleaved into a lower molecular weight active caspase-2. Western blotting analysis showed that the level of procaspase-3 in ZNE-treated capan-2 cells was significantly reduced (1 versus 0.6 ± 0.07-fold reduction). This suggests that ZNE likely induces apoptosis in these cells (Figure 2a).

To further confirm apoptosis in ZNE-treated PC cells, we measured the activity of active caspase 3/7. Our results shown clearly show a significant activation of caspase 3/7 in ZNE-treated compared to vehicle-treated cells (2.67 ± 0.35-fold increase; Figure 2b). Together, these results strongly suggest that ZNE mediates its anti-pancreatic cancer activities, at least through the activation of the type I programmed cell death (PCD1).

### 2.3. Inhibition of ROS Generation, by N-acetyl-cysteine, Decreased the ZNE-induced Cell Death of Capan-2 Cells

**Figure 3:**
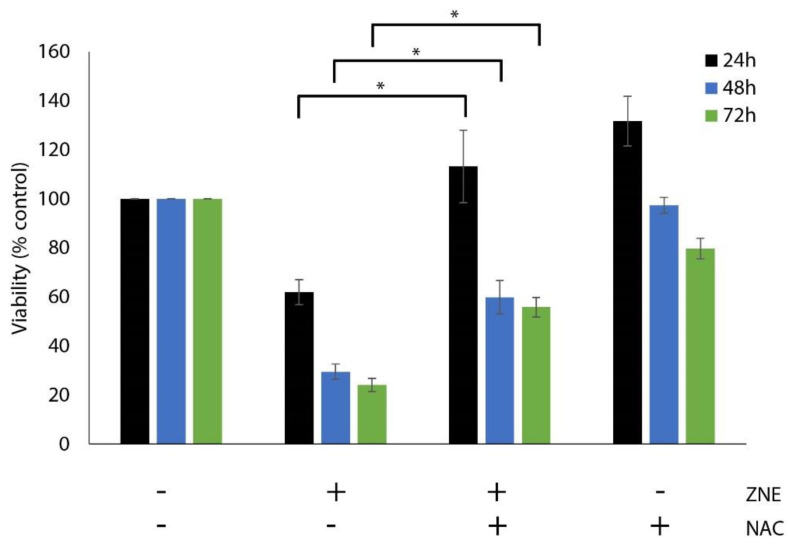
**N-acetyl-cysteine decrease the anti-proliferative effect of ZNE on Capan-2 cells.** Cells were pretreated for 30 min with or without NAC (5 mM) and then with ZNE 200 μg/mL. Then, cell viability was measured using the MTT assay at the indicated time points. Data represent the mean of three independent experiments performed in triplicate and expressed as percentage of the corresponding vehicle-treated control value. In the ZNE and ZNE/NAC, significant differences were noted between bars with similar colors (* denotes a *p* < 0.05).

Oxidative stress is known to affect many aspects of cancer cell behavior, implicating a delicate balance of reactive oxygen species (ROS) and antioxidant protein levels [16,17]. To investigate whether ZNE exerts its anti-proliferative effects through the generation of ROS, cells were pretreated first with the ROS inhibitor, N-acetyl cysteine (NAC), prior to treatment with ZNE. As shown in Figure 3, NAC significantly rescued ZNE-mediated cell death (Figure 3). For instance, the viability of cells treated with ZNE for 24 h was 61.9 ± 5.1% or 113.2 ± 14.8% in the absence or presence of NAC. Thus, our results suggest that ZNE exerts its anti-proliferative effect in PC cells through an ROS-dependent mechanism.

### 2.4. ZNE Inhibits Migration of Capan-2 Cells

**Figure 4:**
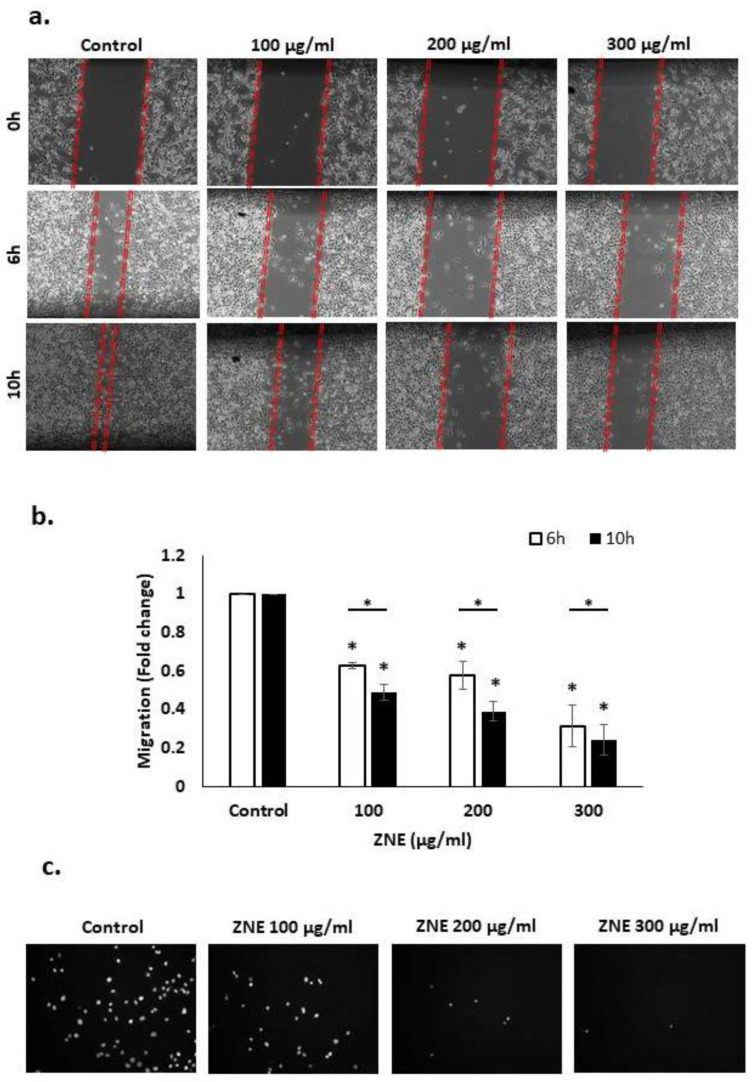
**Ziziphus nummularia inhibits the migration of Capan-2 cancer cells.** (**a**) Confluent cultures of Capan-2 cells were wounded by scratching with a pipette tip, and the cells were incubated without and with the indicated concentrations of ZNE. Representative photomicrographs of the wound were taken at the indicated timepoints. (**b**) Values are plotted as fold change of the vehicle-treated (control) cells. Data represent the average of three replicates. Two-way ANOVA shows that the ZNE-repressed migration is statistically significant in a concentration and time-dependent fashion. (**c**) Capan-2 cells were incubated for 24 h with or without the indicated ZNE concentrations in Boyden chamber transwell inserts as described in Materials and Methods. Migrating Capan-2 cells were stained with Nucblue^®^ and photographed at ×10 magnification. (* denotes a *p* < 0.05).

Cell migration, invasion, and adhesion are key hallmarks of the cancer malignant phenotype. Specifically, cell migration is a crucial process in many biological processes such as tissue formation, wound repair, and proper immune response. However, the deregulation of this process can lead to cancer metastasis, whereby cells disseminate from the primary tumor site and spread to other organs, resulting in their colonization. Here, we tested the effect of ZNE on the migration of Capan-2 cells by the scratch and transwell migration assays. Wound-healing assay revealed that the ability of Capan-2 to close the wound was inhibited by ZNE in a concentration- and time-dependent manner (Figure 4a,b). For instance, 6 h after the cell monolayer was scratched, the migration of cells treated with 100, 200, or 300 μg/mL was 0.63 ± 0.01, 0.58 ± 0.05, or 0.32 ± 0.08 fold that of vehicle-treated (control) cells. Importantly, at the timepoints shown (6 and 10 hrs), ZNE did not cause any significant inhibition of viability of these cells (data not shown), suggesting that the inhibited migration is not due to decreased viability. These results were even more robustly visible in transwell migration chamber assays. Indeed, therein, ZNE caused a significant and dramatic decrease in the cell migration ability of Capan-2 cells to cross from the upper to lower chambers (Figure 4c).

### 2.5. ZNE Inhibits the Invasive Potential of Capan-2 through Downregulating MMP-9

**Figure 5:**
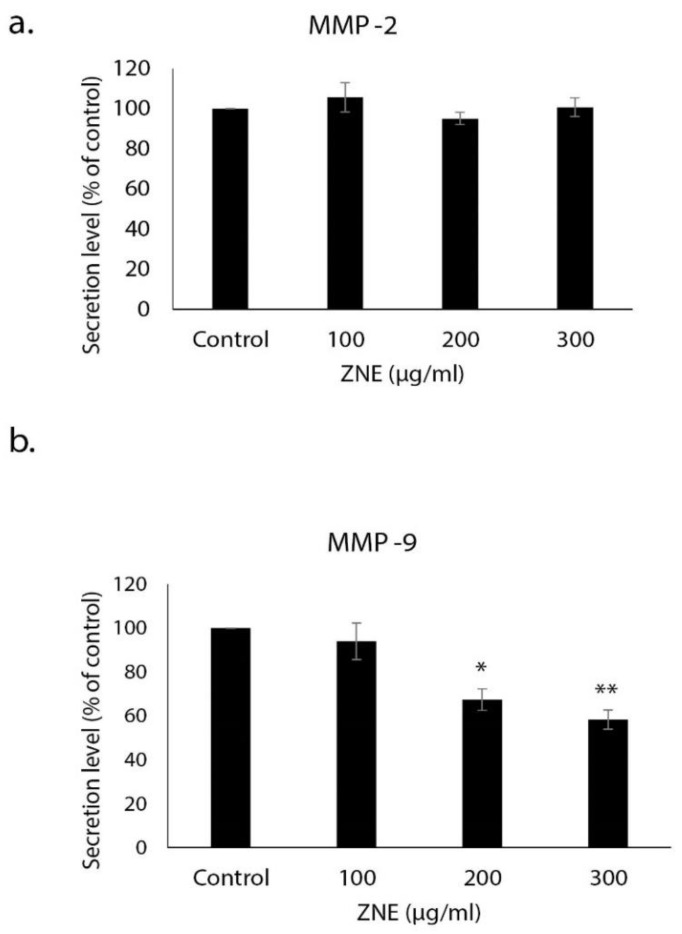
***Ziziphus nummularia* inhibits MMP-9 secretion.** Capan-2 cells were grown in the presence or absence of ZNE. Levels of secreted (**a**) MMP-2 and (**b**) MMP-9 in the conditioned medium of ZNE-treated cells were determined by ELISA. Data represent the mean ± SEM (*n* = 3 replicates) from three independent experiments. * denotes *p* < 0.05 and ** *p* <0.01.

A critical feature of cancer progression and metastasis is the ability to spread away from the primary tumor site and invade other tissues. Toward this, degradation of the extracellular matrix (ECM) around the primary tumor by proteolytic enzymes such as matrix metalloproteinases (MMPs) is a crucial step. We investigated the effect of ZNE on the levels of MMP-2 and MMP-9 in the conditioned medium obtained from control and ZNE-treated Capan-2 cells. Our results showed that ZNE significantly decreased the levels of secreted MMP-9 but not that of MMP-2 (Figure 5). Indeed, the level of MMP-9 in cells treated with 100, 200, or 300 μg/mL of ZNE was 94.0 ± 8.3, 67.3 ± 4.9, or 58.3 ± 4.4% that of vehicle-treated (control) cells. As such, our data suggest that ZNE might inhibit the invasive capacity of Capan-2 at least through downregulation of MMP-9 expression.

### 2.6. ZNE Decreases Adhesion of Capan-2 to Collagen and Downregulates the Expression of Integrin α_2_

**Figure 6:**
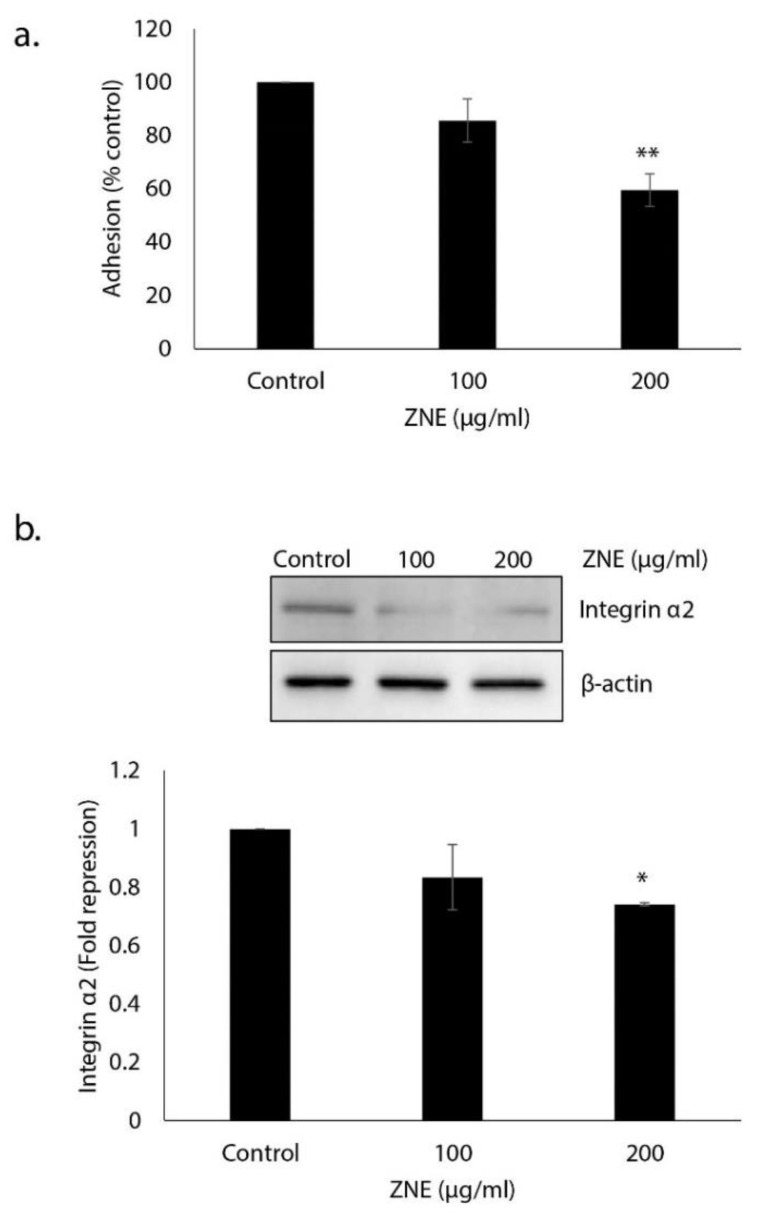
***Ziziphus nummularia* inhibits the adhesion of Capan-2 cells to collagen and downregulates α_2_ integrin.** (**a**) Capan-2 cells were treated overnight with or without the indicated concentrations of ZNE and then seeded onto collagen-coated wells and allowed to adhere for 10 min as described under Materials and Methods. Adhesion was assessed by MTT assay and determined as a percentage of the corresponding vehicle-treated control value. Data represent the mean ± SEM (*n* = 3 replicates) from three independent experiments. ** denotes *p* < 0.01. (**b**) Capan-2 cells were incubated for 48 h with or without the indicated concentrations of ZNE, and whole cell extracts were subjected to Western blotting analysis for integrin α_2_ expression using β-actin as loading control. Data represent the mean ± SEM from three independent experiments. * denotes *p* < 0.05.

The adherence of tumor cells to the extracellular matrix (ECM) is a prerequisite for the invasion and proliferation of many cells, and it is therefore associated with increased metastatic potential. To this end, we tested the effect of ZNE on the adhesive ability of Capan-2 to collagen, which is an ECM protein. Figure 6a shows that pretreatment with ZNE significantly reduced the adhesion of Capan-2 to collagen. Indeed, adhesion in cells treated with 100 or 200 μg/mL of ZNE was 85.6 ± 8.1 or 59.6 ± 6.1% that of vehicle-treated (control) cells.

Cell adhesion to and migration through the ECM is mediated by a family of adhesion molecules, the majority of which are integrins. It is well known that the altered expression of integrin subunits is associated with an increase in the metastatic potential of cancer cells. Studies showed that the α1-, α2, α3-, and α6-integrin subunits were upregulated in pancreatic adenocarcinoma [18]. Here, we show that ZNE (200 μg/mL) caused a significant downregulation in the expression level of the α2 integrin in Capan-2 cells (Figure 6b). Altogether, our results strongly suggest that ZNE could inhibit the migration and invasion capabilities of Capan-2 through disruption of the integrin–ECM axis.

### 2.7. ZNE Increases Aggregation of Capan-2

**Figure 7:**
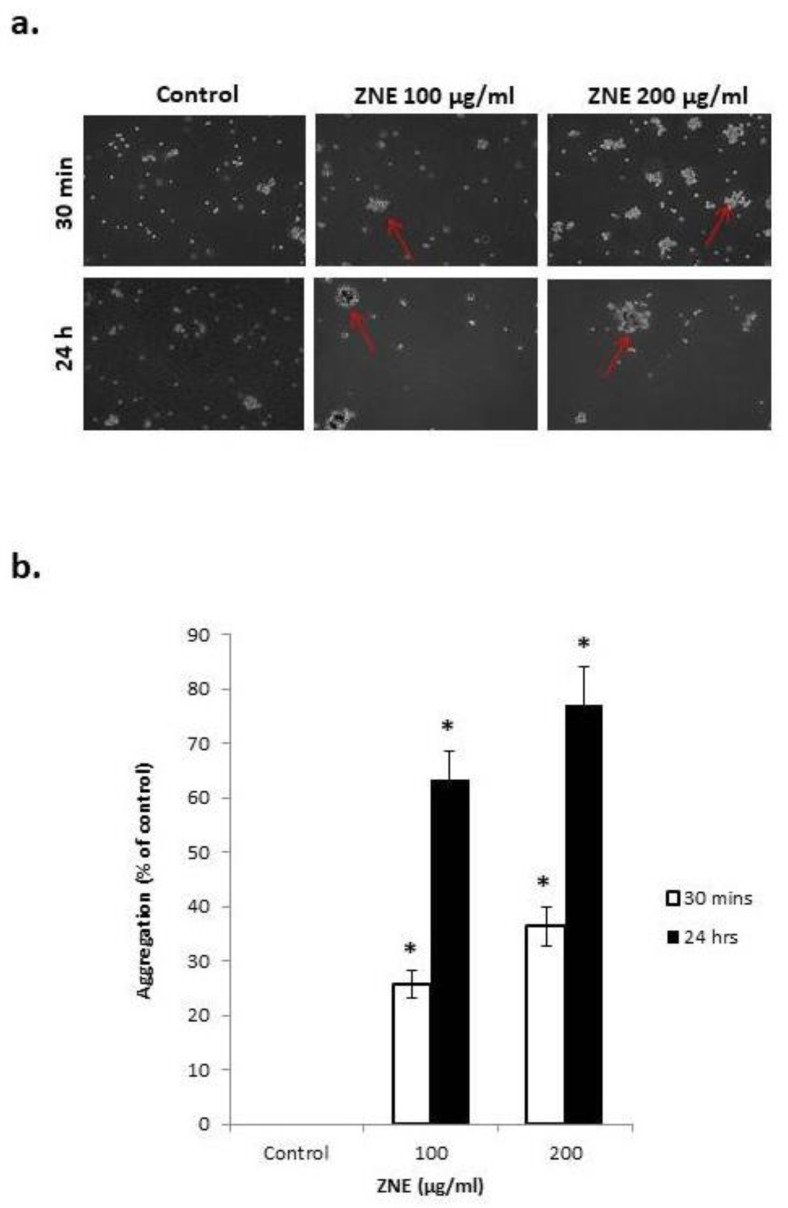
***Ziziphus nummularia* promotes cell–cell aggregation of Capan-2 cells.** Capan-2 cells were incubated without or with the indicated concentrations of ZNE and subjected to cell aggregation as described in Materials and Methods. (**a**) Micrographs of the cells were then taken at 10× magnification. (**b**) Percentage of aggregation was calculated using the following equation: % aggregation = (1 − Nt/Nc) × 100, where Nt and Nc represent the number of single cells in ZNE-treated or control groups, respectively. * denotes a *p* < 0.05, compared to control values at the respective times.

Having shown that ZNE reduces cell–matrix interaction, we next sought to examine the effect of this extract on the cell–cell adhesion of Capan-2 cells in suspension. Toward this, a cell aggregation assay was performed, where Capan-2 cells were incubated, while shaking in a non-adherent plate, in the presence or absence of ZNE. Figure 7 shows that ZNE caused a concentration- and time-dependent increase in cell–cell aggregates compared to control. Indeed, 30 min after incubation with 100 or 200 μg/mL of ZNE, there was a 25.8 ± 2.6 or 36.4 ± 3.6% increase in aggregation, respectively. This percentage increased to 63.4 ± 5.3 or 77.2 ± 6.9% after 2 h of ZNE treatment. Interestingly, this aggregation was concomitant with an increased transcription of E-cadherin (data not shown), which is a protein known to play vital roles in promoting cell–cell adhesion and suppressing malignancy.

### 2.8. ZNE Reduces VEGF Production in Capan-2 and Inhibits Angiogenesis in Ovo

**Figure 8:**
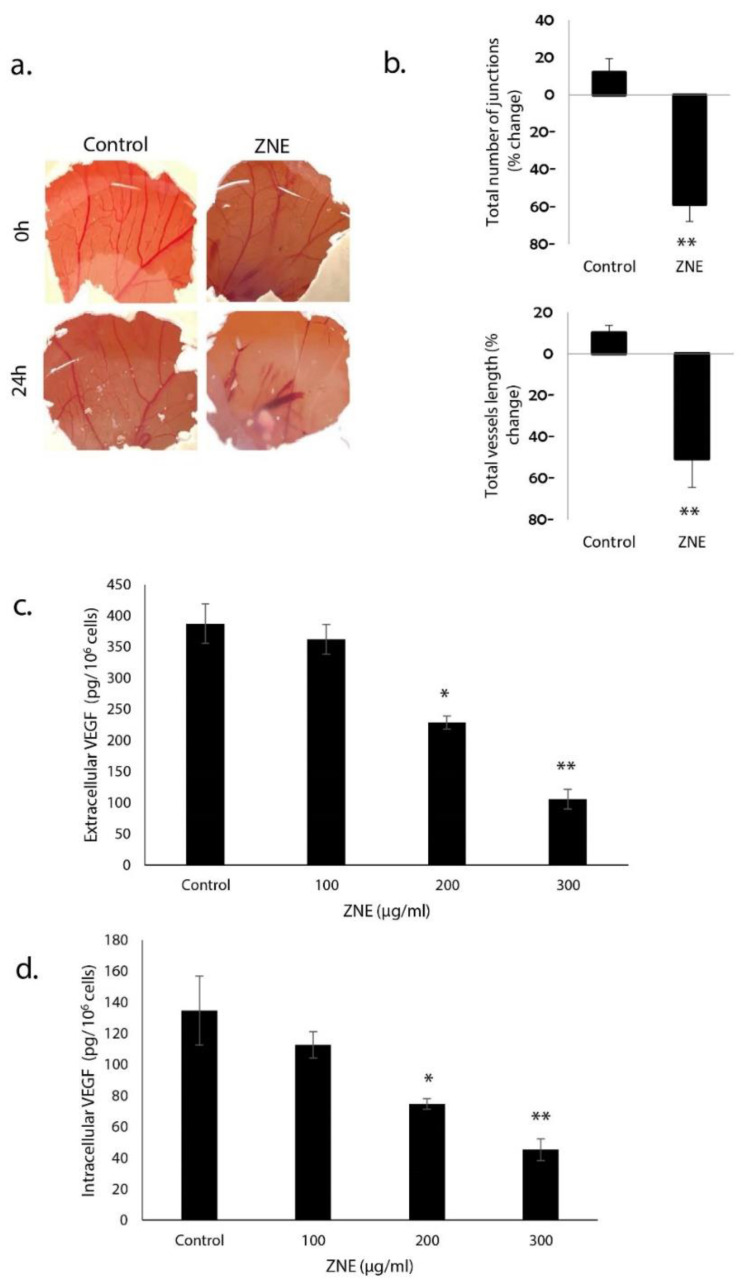
***Ziziphus nummularia* inhibits angiogenesis in ovo and reduces extracellular and intracellular VEGF levels in Capan-2 cells.** (**a**) ZNE was applied on the CAM of fertilized eggs as described in Materials and Methods, and the angiogenic response was scored by taking pictures of the CAM after 24 h. (**b**) Percentage changes in total vessel length and total number of junctions, for both control and ZNE-treated CAMs, were quantified using the AngioTool software. (**c**) Cells were treated with or without the indicated concentrations for ZNE for 24 h, and the levels secreted VEGF in the conditioned medium were analyzed by ELISA as described under Materials and Methods. (**d**) Similarly, levels of intracellular VEGF were analyzed by ELISA. Data represent the means ± SEM of three independent experiments. * denotes *p* < 0.05 and ** *p* < 0.01.

Tumor growth and metastasis mainly depend on angiogenesis; therefore, inhibiting this process would inhibit tumor expansion and metastasis. The effect of ZNE on angiogenesis was examined in ovo using the chick embryo chorioallantoic membrane (CAM) assay. ZNE was applied onto the surface of the highly vascularized CAM membrane, and the embryo was replaced in the egg hatcher for another 24 h. As shown in Figure 8a, ZNE treatment caused a significant inhibition of new blood vessel formation as well as the number of junctions. Importantly, with 200 μg/mL of ZNE for 24 h, both the total length of vessels and the total number of junctions were significantly decreased by 50.8 ± 7.3% or 58.7 ± 9.6%, respectively (Figure 8b).

The anti-angiogenic potential of ZNE was further investigated by testing its effect on the production of secreted and intracellular vascular endothelial growth factor (VEGF), which is a pro-angiogenic protein critically required for blood vessel formation. We found that ZNE significantly reduced, in a concentration-dependent manner, the level of both secreted (Figure 8c) and intracellular (Figure 8d) VEGF. Indeed, the amount of extracellular VEGF (in pg/10^6^ cells) released from cells treated with vehicle (control), or 100, 200, or 300 μg/mL of ZNE was 387.7 ± 31.8, 362.6 ± 23.9, 229 ± 10.2, or 106 ± 15.5. Similarly, the amount of intracellular VEGF (in pg/10^6^ cells) in cells treated with vehicle (control), or 100, 200, or 300 μg/mL of ZNE was 134.7 ± 22.2, 112.7 ± 8.4, 74.7 ± 3.4, or 45.3 ± 7.0. It is noteworthy to mention that the role of intracellular VEGF is not limited but can also contribute to other tumorigenic events including tumor initiation, tumor growth, migration, and invasion [19]. Importantly, ZNE also significantly suppressed the production of nitric oxide, which is another potent angiogenic factor (data no shown).

### 2.9. ZNE Inhibits ERK1/2(MAPK) and NFκB Signaling Pathways

**Figure 9:**
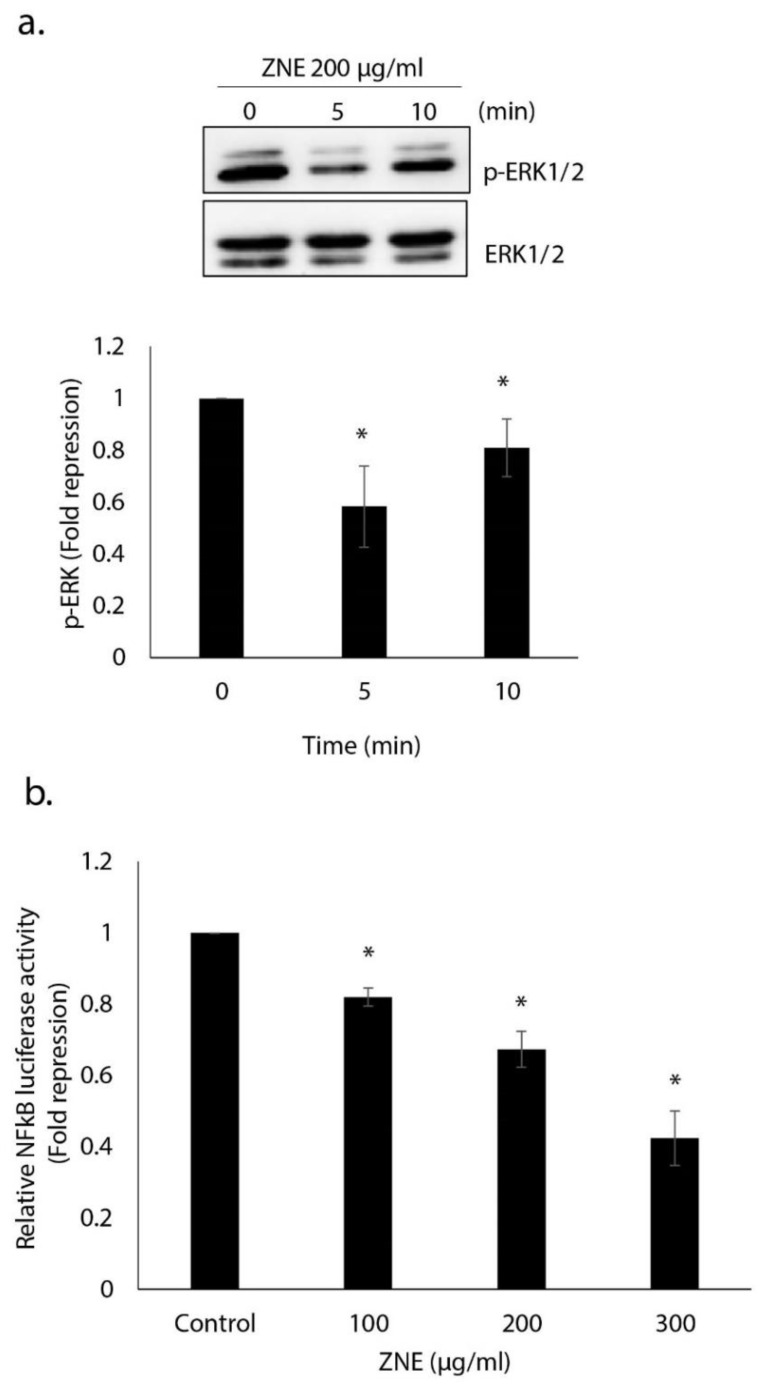
***Ziziphus nummularia* inhibits the ERK1/2 and NFκB signaling pathways in pancreatic cancer cells.** (**a**) *Ziziphus nummularia* downregulates the level of phosphorylated ERK1/2. Cells were treated without or with ZNE (200 μg/mL) for 5 and 10 min, and protein lysates were examined for the level of phosphorylated ERK1/2. Data are representative of three independent experiments. (**b**) *Ziziphus nummularia* inhibits NF-κB transcriptional activity. Capan-2 cells were transfected with the pGL4.32[luc2P/NF-κB-RE/Hygro] expression plasmid, and luciferase activity was measured 18 h post-transfection as described in Materials and Methods. Data represent the means ± SEM of three independent experiments. * denotes *p* < 0.05.

Overwhelming evidence shows that ERK1/2 is a master orchestrator of cell cycle progression, and its activation is intimately associated with increased growth, proliferation, and regeneration [20]. When activated by RAF (RafA, RafB, and c-Raf1) or MEK (1 and 2) via the canonical RAF–MEK–ERK pathway or by EGF and CCK [21,22], ERK potentiates cytokine production by pancreatic acinar cells [23,24]. In PC, ERK is constitutively activated, and approaches geared toward downregulating it are indeed used as attractive avenues in the management of treatment [25]. Here, we report that ERK1/2 activation in cells treated with ZNE for 5 min was 0.58 ± 0.1-fold lower than in vehicle-treated (control) cells (Figure 9a).

NF-κB is a group of transcription factors that are activated in response to radiation, stress, and exposure to cytokines [26]. In cancer, NF-κB interacts with a plethora of factors that eventually promote cancer development and progression [27,28]. In PC, NF-κB is involved in cell proliferation, invasion, and angiogenesis [29]. In fact, ERK activation induces NF-κB nuclear accumulation and transcriptional activity [30]. Furthermore, inhibition of the ERK/NF-κB signaling pathway attenuates PC migration and invasion [31]. To test the effect of ZNE on this pathway, we measured the NF-κB transcriptional activity by transiently transfecting Capan-2 cells with an NF-κB-driven luciferase reporter expression vector. Results showed that ZNE repressed NF-κB-dependent transcription in a concentration-dependent manner (Figure 9b). Indeed, the NF-κB of cells treated with 100, 200, or 300 μg/mL was 0.82 ± 0.02, 0.67 ± 0.05, or 0.42 ± 0.08 fold that of vehicle-treated (control) cells.

## 3. Discussion

More than 3000 plant species contain cytotoxic compounds such as alkaloids, flavonoids, terpenoids, and saponins [32,33]. Plants bountiful in such compounds are considered to have promising anticancer potential. Specifically, studies indicate that plants belonging to *Ziziphus* species present potent anti-tumorigenic properties [34]. For instance, *Zizyphus mauritiana*, *Zizyphus rugosa*, and *Zizyphus oenoplia* have selective cytotoxicity against human melanoma cell lines [35]. In addition, *Zizyphus mauritiana* attenuated the proliferation of human lung carcinoma A549 cells and *Zizyphus oenoplia* inhibited the survival of human MDA-MB-231 triple negative breast cancer cells [36].

Here, we showed that the ethanolic extract of *Z. nummularia* possesses anti-proliferative, anti-migratory, and anti-invasive effects on the PC cells, Capan-2. These anti-tumorigenic events were concomitant with a decrease in the activity of ERK1/2 MAPK as well as in the expression of α_2_ integrin and MMP-9. Furthermore, ZNE reduced VEGF secretion and NO production in Capan-2 cells as well as attenuated in ovo angiogenesis. Additionally, downregulation of the transcriptional activity of NF-κB suggests that ZNE mediates its anticancer effects also through inhibiting the NF-κB pathway, which is a major pro-inflammatory pathway implicated in the carcinogenesis of many cancer types. These results support the anticancer potential of ZNE and present *Z. nummularia* as a potential natural supplement against PC, provided that further investigations validate its in vivo effect and ensure its safety. To our knowledge, this is the first study to assess the effect of *Z. nummularia* on the tumorigenic phenotype of PC cells.

Overwhelming evidence indicates that tumorigenic cells become highly proliferative [37]. Consequently, inhibiting this uncontrolled proliferation is a key step in cancer treatment. In our study, we showed that ZNE attenuated the proliferation of Capan-2. This is in line with the cytotoxic effect of *Z. nummularia* on five cancer cell lines: human breast cancer (MCF-7), leukemia (K-562), ovarian cancer (OVCAR-3), human colon adenocarcinoma (HT-29), and human kidney carcinoma (A-498) [13]. However, this effect was not imparted by *Z. nummularia* leaf extract but rather by a compound isolated from *Z. nummularia* root barks [13]. While an isolated compound may provide some advantages, it is not always the optimum choice given that synergy between various bioactives often dictates the ultimate therapeutic value of a plant. Moreover, from an ethno-medicinal perspective, people consume the whole leaf and thus, the crude extract provides more relevance. Nonetheless, it is important to mention here that bio-guided fractionations as well as identifying and purifying specific bioactives remain an important ethnopharmacological approach. Relevantly, other *Ziziphus* plants showed similar anti-proliferative effect. For example, *Z. spina-christi* showed significant cytotoxic effects on HeLa and MAD-MB-468 cells [33]. In addition, *Ziziphus jujube* exhibited cytotoxic effects against HeLa [38], MCF-7 [39], SKBR3 [39], and MDA-MB-468 cells [40], the Jurkat leukemia cell line, and HEP-2 human larynx carcinomas cells [41].

Reactive oxygen species (ROS) are key signaling molecules implicated in both pro- and anti-tumorigenic effects [42]. In our study, the pharmacological inhibition of ROS attenuated the anti-proliferative effect of ZNE in Capan-2 cells. This suggests that ZNE induces an increase in intracellular ROS, which in turn inhibits cell proliferation. This is in accordance with a previous report showing that another *Ziziphus*, namely *Ziziphus jujuba*, induced ROS in human hepatoma cells (HepG2) [43]. Interestingly, this increase in ROS levels was concomitant with decreased cell viability [43]. Contextually, betulinic acid, a bioactive compound found in *Ziziphus jujube*, can trigger ROS production [44].

High ROS levels are permissive and rather augment a high proliferative rate in cancer cells [45]. However, the effect of ROS is dose-dependent [45]. Several studies reported that certain plant extracts induce cytotoxicity in cancer cells by blocking cellular antioxidant defense systems [46,47,48,49]. In addition, chemotherapy and radiotherapy eradicate cancer cells by increasing intracellular ROS [50]. In our study, pretreatment with ROS inhibitor, N-acetyl cysteine (NAC), significantly rescued ZNE-mediated cell death. Thus, ZNE exerts its anti-proliferative effects through the generation of ROS. However, further experiments aimed at determining oxidative biomarkers are warranted to further validate this notion.

It worth mentioning that oxidative homeostasis is greatly modulated by the transcription factor NF-E2 Related Factor-2 (NRF2) [51]. Indeed, NRF2 regulates the expression of many genes involved in cellular defense against oxidative stress. Interestingly, NRF2-activating drugs are considered to be effective treatments for oxidative stress diseases such as cancer [51]. In addition, certain plant extracts and plant-derived compounds were shown to activate NRF2, thus exerting a cytoprotective effect [51,52]. Furthermore, the transcription of antioxidant genes is upregulated in the events of stabilizing mutations in gene encoding NRF2 or inactivating mutations in its negative regulator (Keap1) [53,54]. Consequently, Nrf2 is stabilized and accumulated in response to oxidative stress. However, some studies reported a cancer-promoting effect induced by NRF2 hyperactivation. For instance, Nrf2 activation promoted lung cancer metastasis [55]. This is achieved by mutations in the Keap1–Nrf2 axis [55]. In accordance, the administering ROS inhibitor (NAC) the antioxidant vitamin E to mice with lung cancer promoted cancer metastasis by the same mechanism [56]. It seems that the regulation of genes encoding NRF2 is cell type-dependent [57]. In our study, ZNE attenuated the cancer cell proliferation in an ROS-dependent manner. It may be tempting to speculate that ZNE-induced ROS leads to these NRF-2-stabilizing mutations.

Overwhelming evidence shows that natural compounds exert pro- or anti-oxidant effect, depending on several factors including concentration, redox potential, the presence of metal ions, as well as the cell type or animal model [58,59,60]. Contextually, overwhelming evidence supported the antioxidant potential of *R. rosea* [61,62], *R. canina* [63,64], *H. perforatum* [65,66], and *G. lutea* [67,68]. However, their anti-oxidant capabilities are reduced as the plant extract concentration increases [69]. In addition, environmental conditions such as pH are known to affect the oxidative capacity of natural compounds. Indeed, it was shown that akaline pH decreases the in vitro anti-oxidant activity [70]. Similar results were obtained in vivo where some plant extracts exert an anti-oxidative effect at acidic but not alkaline pH [70].

Evidence also indicates that certain conditions may drive certain known antioxidants to assume a pro-oxidant capacity. For instance, polyphenols and vitamins C and E, molecules well-documented for their antioxidant activity, sometimes elicit a pro-oxidant effect [71]. The interaction of polyphenols with transition metal ions leads to a pro-oxidant effect [72,73,74]. Similarly, vitamin E becomes a pro-oxidant at high concentrations [71]. The well-known anti-oxidant vitamin C plays a pro-oxidant role at high concentrations. Indeed, while vitamin C possesses an anti-oxidative potential at low doses (30–100 mg/kg body weight), it plays a pro-oxidative role at high doses (1000 mg/kg body weight) [75,76,77]. In addition, vitamin C shows a pro-oxidant effect in the presence of iron (Fe^3+^) or copper (Cu^2+^) [73,74]. Furthermore, the combination of vitamin C and Trolox (water-soluble analog of vitamin E) may provoke mild oxidative stress [78]. Another known anti-oxidant, α-tocopherol, becomes a pro-oxidant at high concentrations [71]. Taken together, these studies show that the dose or the microenvironment play critical roles in determining the pro- versus anti-oxidative effect of a given molecule.

Flavonoids have also been reported to act as pro-oxidants under conditions that favor their auto-oxidation such as the presence metal ions and alkaline pH transition metals [79,80]. Another factor that determines the anti-/pro-oxidative potential of flavonoids is their medium. For instance, the flavonoid quercetin is mutagenic in bacterial test systems, which is probably due to its oxidative potential [81]. Gossypol, a polyphenol isolated from the cotton plant, is an anti-oxidant [82,83]. However, it plays a pro-oxidant role in vitro [84] and in the presence of liver microsomes [82]. Furthermore, the administration of high doses of the polyphenol, epigallocatechin gallate (EGCG), lead to some oxidation in vivo [85]. In vitro, EGCG oxidizes immediately and induces cytotoxic oxidative levels [85]. This may be caused by the metabolites resulting from rapid flavonoids metabolism [79].

It is well-established that cancer cells lose their connection with extracellular matrix (ECM) as they acquire increased migratory and invasive capacities, both of which are crucial for metastasis. In addition, cancerous cells lose cell–cell adhesion, significantly contributing to uncontrolled cell proliferation, further promoting tumor growth and dissemination [86]. Our results demonstrated that ZNE attenuated the adhesion, migration, and invasion of PC cells, and it increased their aggregation. While the vast majority of studies assessing the anticancer potential of *Ziziphus* plant extracts were limited to their cytotoxic effects [33,38], this is the first study reporting attenuated cellular migration and invasion by a species of *Ziziphus*; hence, the significance of our findings. Interestingly, a bioactive compound of *Z. lotus* extract, protopheophorbide A, was shown to attenuate cell adhesion of the breast cancer cell line, MDA-MB-231 [87]. Additionally, our study shows that ZNE induced a decrease in α_2_ integrin expression. This downregulation is anticipated to mediate a ZNE-induced decrease in adhesion. Whether the modulation of other integrins is involved in this anti-adhesive effect remains to be tested.

**Figure 10:**
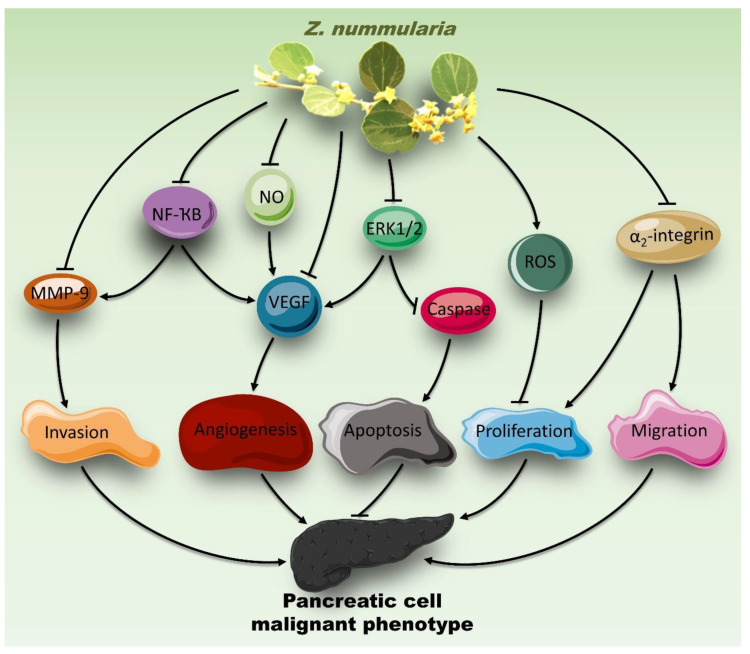
Cascade of the mechanism of action of *Ziziphus nummularia* in Capan-2 cells.

The mitogen-activated protein kinase ERK1/2 is a major regulator of various cellular processes including cell proliferation and migration [88]. The activity of ERK1/2 is known to be upregulated in tumors [88]. Therefore, compounds targeting ERK1/2 present advantages when designing drugs for the management or treatment of cancer. Here, we showed that ZNE attenuated the phosphorylation of ERK1/2. This inhibition is assumed to underlie the ZNE-induced anti-proliferative, anti-migratory, anti-invasive, and anti-angiogenic effects (Figure 10) given that ERK1/2 is overwhelmingly documented to promote these phenotypes in PC cells. Our result is in accordance with previous studies reporting that *Ziziphus lotus* attenuated the proliferation of immortalized Jurkat T-cells by inhibiting ERK1/2 [89].

The ability of cancer cells to disseminate from the primary tumor is a crucial step in cancer invasion and metastasis. These processes involve the degradation of the ECM by proteases such as members of the matrix metalloproteinase (MMP) family. Indeed, increased levels of MMPs are associated with increased tumor growth and metastasis. In particular, MMP-2, MMP-7, and MMP-9 are considered as malignant markers for PC [90]. In fact, serum levels of MMP-9 are not only indicative of the prognosis of patients with PC but are also directly involved in cancer progression [91,92]. Indeed, our results showed that ZNE exhibits its anti-invasive effect by reducing ECM degradation through downregulating MMP-9 levels in Capan-2 cells.

Angiogenesis, the formation of new blood vessels, is a crucial process required to supply proliferating and metastatic tumor cells with nutrients and other humoral factors. Our results show that ZNE exhibited an anti-angiogenic effect. This indeed mirrors a study that showed bioactive compounds in *Z. jujube* exhibiting significant anti-angiogenic potentials [93]. As such, it was proposed that these *Ziziphus* plants can be used as adjuvants along with standard cancer therapies [93]. However, the ethanolic extract of *Z. oenoplia* root increased the number of capillaries on the chick chorioallantoic membrane of 9-day-old fertilized chick eggs [94], suggesting that *Z. oenoplia* possesses an angiogenic potential. This may indicate that the effect of *Ziziphus* on angiogenesis may be species-dependent. Inhibiting angiogenesis can be achieved through targeting pro-angiogenic factors, the most prominent of which are VEGF and NO. Indeed, the high expression of VEGF is associated with poor prognosis of PC [19,95]. Here, we showed that ZNE suppressed levels of VEGF and NO in Capan-2 cells, suggesting that *Z. nummularia* may inhibit tumor growth and metastasis through blocking angiogenesis. Additionally, our data showed that the effect of ZNE is not only limited to the secretion of VEGF but also affected its intracellular levels. Interestingly, VEGF signaling plays a major role in tumor cells, independently of angiogenesis, and is associated with tumor initiation and oncogenesis [19]. Specifically in PC, a “VEGF-trap” that sequesters VEGF has been reported to inhibit tumor growth [96].

Understanding oncogenic signaling pathways is crucial in the development of new therapeutic strategies against cancers. Numerous studies have implicated the role of the transcription factor NF-κB in the development and progression of different cancer types [97]. Importantly, the activation of NF-κB has been observed in PC [28]. Here, we showed that ZNE inhibited the NF-κB pathway by downregulating the transcriptional activity of NF-κB, which is concomitant with the downregulation of its downstream targets mentioned above (MMP-9, VEGF, and NO) (Figure 10). Therefore, the inhibition of the NF-κB pathways could account for the anti-proliferative and anti-metastatic effect of *Z. nummularia*.

*Z. nummularia* contains many bioactive compounds such as tannins, flavonoids, steroids, glycosides, ascorbic acid, pectin-A, thiamine, and alkaloids [98,99]. Owing to its bountiful bioactives, *Z. nummularia* may be an attractive plant for drug discovery. While some of these bioactives may be involved in the anticancer effects of ZNE, further experiments are needed to establish this assumption. It would be interesting to evaluate the effect of *Z. nummularia* in an in vivo model of PC, which is indeed a limitation of this study. Another limitation of our work is the lack of a normal pancreatic cell line that can be used to validate the selectivity of ZNE. However, it is worth mentioning that the selective cytotoxic activity of extracts of various ziziphus species has been documented. For instance, the ethanolic extract of jujube seeds selectively induced Jurkat cell death, which is an effect not reported in normal Vero cells [100]. Similarly, seeds of *Ziziphus mauritiana* showed anti-proliferative effects on HL-60, HeLa, and Molt-4 cell lines without affecting the normal HGF cell line [101,102] or normal rat liver cells [103]. Furthermore, chloroform-based extract of *Ziziphus jujuba* pulp showed selective cytotoxic effects on MCF-7 and SKBR3 cells without affecting normal cells [101]. In addition, ongoing investigations in our laboratory are being conducted to isolate and characterize the bioactive molecules in *Z. nummularia* ethanolic extract.

## 4. Materials and Methods

### 4.1. Cell Culture

Capan-2 human pancreatic cancer cells (CLS Cell Line Service, Eppelheim, Germany) were maintained in a humidified (37 °C and 5% CO_2_) chamber in DMEM high-glucose medium supplemented with 10% fetal bovine serum (FBS) (both from Sigma-Aldrich, St. Louis, MO, USA) and 1% penicillin/streptomycin (Lonza, Switzerland).

### 4.2. Ziziphus Nummularia Extract (ZNE)

Leaves of *Ziziphus nummularia* were rinsed and air dried in the dark at room temperature. Then, they were ground to a fine powder and suspended in 70% aqueous ethanol for 72 h in the dark. Then, the solution was filtered and evaporated to dryness by vacuum distillation. Then, the obtained residue was dissolved in 70% ethanol at a concentration of 200 mg/mL and kept in the dark at 4 °C.

### 4.3. Cell Viability Assay

Capan-2 cells (5 × 10^3^) were seeded in 96-well plates and allowed to grow until they reached 30–40% confluency. Then, cells were treated with increasing concentrations of ZNE and incubated for 24, 48, and 72 h. The viability of the cells was assessed by 3-(4,5-dimethylthiazol-2-yl)-2,5-diphenyltetrazolium bromide (MTT; Sigma-Aldrich, St. Louis, MO, USA) reduction assay. Cell growth was determined as the proportional viability of the treated cells in comparison with the untreated ones, the viability of which was assumed to be 100%. Cell viability assays with N-acetyl cysteine (NAC; Sigma-Aldrich, St. Louis, MO, USA) were carried out by treating the cells with 5 mM NAC solution for 30 min prior to adding ZNE. Assays were performed in triplicate and repeated three times. Data are presented as mean values ± SEM.

### 4.4. Wound-Healing Assay

Capan-2 cells were grown in 12-well plates until confluency. A wound scratch was made through the confluent monolayer using a sterile 200 µL pipette tip. Then, the culture medium was removed, and wells were washed twice with phosphate-buffered saline (PBS; Sigma-Aldrich, St. Louis, MO, USA) to remove cellular debris. Fresh medium in the presence or absence of the indicated concentrations of ZNE was added, and cells were further incubated at 37 °C. Photomicrographs were taken at baseline (0 h), 6 and 10 h timepoints using an inverted microscope (objective 10×). The width of the wound was expressed as the average ± SEM between the measurements taken at time zero and the corresponding time points. Assays were repeated three times, and data were presented as mean ± SEM.

### 4.5. Transwell Migration Chamber Assay

The ability of cells to migrate was also evaluated using transwell inserts (8 μm pore size). Cells were seeded onto the upper chamber of the insert at a density of 4 × 10^4^ cells in 0.1% FBS-containing medium in the presence or absence of ZNE. Regular DMEM medium (supplemented with 10% FBS) was added to the lower chamber in order to act as chemoattractant. Cells were incubated at 37 °C and allowed to migrate for 24 h. Following incubation, cells were washed and fixed with 4% formaldehyde. Non-migrated cells on the upper side of the insert were removed with a sterile cotton-tipped applicator, while cells on the underside of the insert were mounted with anti-fading agent and viewed using the Olympus IX 71 inverted microscope. Assays were repeated three times and data were presented as mean values ± SEM.

### 4.6. Adhesion Assay

Cells were grown in the absence or presence of ZNE for 24 h and then seeded onto collagen-coated 24-well plates in duplicates. Cells were allowed to adhere for 10 min at 37 °C. Then, media was removed, and cells were gently washed off with PBS twice in order to remove non-adhering cells. The number of adherent cells was determined by the 3-(4,5-dimethylthiazol-2-yl)-2,5-diphenyltetrazolium bromide (MTT) reduction assay, as described above.

### 4.7. Aggregation Assay

Cell aggregation was assessed by harvesting cells from confluent plates using 2 mM EDTA in calcium and magnesium free (CMF)-PBS and aliquoted on fresh empty dishes, with or without ZNE. Cells were incubated at 37 °C with shaking for 30 min or 24 h. Then, cells were fixed with 1% formaldehyde, and pictures were taken using the Olympus IX 71 inverted microscope.

### 4.8. Analysis of Apoptotic Morphological Changes

Morphological changes characteristic of apoptotic cells were observed using a phase contrast inverted microscope. For this, cells were grown in 6-well plates in the absence or presence of different concentrations of ZNE. Pictures were taken after 24 and 48 h at 10×, 20×, and 40× magnifications.

### 4.9. Chorioallontoic Membrane (CAM) Assay

Fertilized eggs were incubated at 38 °C with 60% relative humidity for 7 days. Following incubation, the highly vascularized chorioallantoic membrane (CAM) was dropped by drilling a 1 cm^2^ window though the eggshell into the air sac. The effect of ZNE on blood vessel growth was tested by applying ZNE onto the CAM. The angiogenic response was scored by taking pictures of the CAM 24 h following ZNE incubation, and they were analyzed using AngioTool software in order to quantify the length of the vessels and number of junctions.

### 4.10. Western Blotting Analysis

Cells were washed twice with PBS, scraped, and lysed using 2% SDS, 60 mM Tris lysis buffer (pH 6.8). Then, cell lysates were centrifuged at 5000 g for 10 min. The protein concentration of the supernatants was determined using the Lowry method. Then, aliquots of 25–30 µg were loaded in each lane and separated by 10% sodium dodecyl sulfate-polyacrylamide gel electrophoresis and transferred to a polyvinylidene difluoride membrane (Immobilon PVDF; Biorad, Hercules, CA, USA). Immunodetection was carried out using the specified primary antibody and appropriate secondary antibody. Rabbit monoclonal p44/42 MAPK (ERK1/2), phospho-p44/42 MAPK (ERK1/2) (Thr202/Tyr204), integrin α_2_, and pro-caspase-3 were used at a 1:1000 dilution in PBS-T and incubated overnight at 41 °C. Mouse monoclonal anti-β-actin antibodies, also at 1:1000 dilution, were used for the loading control. All primary and secondary antibodies were obtained from Cell Signaling (Cell Signaling Technology, Inc., Danvers, MA, USA). For quantification, experiments were repeated three times. Data are presented as mean values ± SEM.

### 4.11. Measurement of Caspase 3/7 Activity

Cells were seeded (5000 cells/well), in triplicate, in 96-well plates and treated with the indicated concentrations of ZNE or vehicle (70% ethanol) for 48 h. Levels of capase-3/7 were measured using the luminescent caspase-Glo 3/7 assay kit (Promega Corporation, Madison, WI, USA) following the manufacturer’s protocol. Briefly, caspase reagents were added to the cells and incubated for 2.5 h in the dark at room temperature on an orbital shaker. The luminescent signal was measured using the Berthold FB12 Luminometer. Data were presented as a proportional viability of the treated cells in comparison with the untreated ones, the viability of which is assumed to be 100%. The experiments were repeated 4 independent times.

### 4.12. Measurement of MMP-2 and MMP-9 by ELISA

Cells were seeded in 6-well plates in the presence of vehicle or ZNE for 24 h. Then, the conditioned medium was collected, and the levels of secreted MMP-2 and MMP-9 were determined using ELISA kits (R&D Systems, Minneapolis, MN, USA or Invitrogen, Camarillo, CA, USA), according to the manufacturer’s protocol. The assays were performed in triplicates and repeated three times. Data are presented as mean values ± SEM.

### 4.13. Measurement of Human Vascular Endothelial Growth Factor (VEGF)

Capan-2 cells were seeded in 24-well plates, cultured overnight, and then treated with or without the indicated concentrations of ZNE for 24 h. The conditioned medium was collected, and the level of VEGF was measured using a VEGF enzyme-linked immunosorbent assay kit (R&D Systems, Minneapolis, MN, USA) according to the manufacturer’s instructions. The assays were performed in triplicate and repeated three times. Data are presented as mean values ± SEM.

### 4.14. Luciferase Activity for NF-κB

Capan-2 cells were seeded in 12-well plates and cultured overnight. Then, cells were transfected with the NF-κB luciferase reporter expression plasmid pGL4.32[luc2P/NF-κB-RE/Hygro] and *Renilla* expression plasmid (Promega, Madison, WI, USA) using Fugene HD transfection reagent (Promega, Madison, WI, USA) according to the manufacturer’s protocol. Briefly, transfected cells were incubated overnight, after which the medium was replaced with fresh complete medium with or without increasing concentrations of ZNE. Luciferase activity was measured using the Dual Luciferase Reporter Assay System (Promega, Madison, WI, USA). Firefly luciferase was normalized against the *Renilla* luciferase reporter which was used as an internal control. Experiments were carried out in triplicate and repeated three times, and the average of three means is represented ± SEM.

### 4.15. Statistical Analysis

Data were statistically evaluated using Student’s *t*-test using GraphPad Prism version 5.0. For the comparison of more than two means, ANOVA was used: either one-way ANOVA (with Dunnett’s post hoc test) or two-way ANOVA (with Tukey–Kramer’s post hoc test). Data were presented as mean ± standard error of the mean (SEM). A *p*-value of less than 0.05 was considered as significant.

## Data Availability

All data generated or analyzed during this study are included in this published article.

## Sample Availability

Samples of the compounds are not available from the authors.

## Abbreviations

PC	Pancreatic cancer
ZNE	Ziziphus nummularia extract
ROS	Reactive oxygen species
NAC	N-acetyl cysteine
ECM	Extracellular matrix
CAM	Chorionoallantoic membrane
VEGF	Vascular endothelial growth factor
ERK	Extracellular signal-regulated kinase
MMP	Matrix metalloprotease
